# Post stroke hemi-dystonia in children: a neglected area of research

**DOI:** 10.1186/s40348-015-0026-2

**Published:** 2015-12-11

**Authors:** Daniel Tibussek, Ertan Mayatepek, Dirk Klee, Anne Koy

**Affiliations:** Department of General Pediatrics, Neonatalogy and Pediatric Cardiology, University Children’s Hospital, Heinrich-Heine University, Moorenstrasse 5, 40225 Düsseldorf, Germany; Department of Diagnostic and Interventional Radiology, Faculty of Medicine, University Dusseldorf, Heinrich-Heine University, Moorenstrasse 5, 40225 Düsseldorf, Germany; Department of Neurology, University of Cologne, Kerpener Strasse 62, 50924 Cologne, Germany

**Keywords:** Childhood stroke, Basal ganglia, Dystonia, Movement disorders, Functional imaging, Transcranial magnetic stimulation

## Abstract

**Background:**

Childhood arterial ischemic stroke (CAIS) is increasingly recognized as an important cause of significant long-term morbidity in the pediatric population. Post stroke movement disorders, above all hemi-dystonias, are much more common in children after stroke compared to adults. However, research in this field is largely lacking. By highlighting some important knowledge gaps, we aim to encourage future collaborative research projects in this particular field.

**Findings:**

Post stroke-dystonia seems to be much more common among children than adults. However, no reliable epidemiological data of post-stroke movement disorders in childhood are available, and differentiation between spasticity and dystonia can be challenging. Pharmacotherapy for dystonia is limited by lack of effect, especially in the long-term treatment. The pathophysiology of dystonia is complex and incompletely understood. Recent findings from functional imaging studies suggest that dystonia does not result from a single lesion but rather network dysfunctions and abnormalities in functional connectivity. However, very few patients with post stroke dystonia have been studied, and it is not clear to what extent pathophysiology of primary and post stroke ischemia shares common characteristics on network level. In general, progress in understanding the nature of childhood dystonia lags far behind adult onset CNS diseases.

**Conclusions:**

Dystonia after CAIS is a common yet insufficiently understood and poorly studied clinical challenge. Studies to improve our understanding of the underlying pathophysiology and consequently the development of instruments for early prediction as well as targeted treatment of dystonia should become a high priority in collaborative childhood stroke research.

## Background

Childhood arterial ischemic stroke (CAIS) is an important cause of morbidity and mortality in the pediatric population. With an estimated incidence of 1.6 per 100,000 children per year (excluding neonates; [1]), more than 200 new cases can be expected to occur in Germany each year. Early brain injury due to stroke commonly results in significant long-term impairment [2–5]. Hemi-dystonia is a common, yet insufficiently studied motor problem after CAIS leading to significant life-long disability [6].

The purpose of this review is to summarize current knowledge about post stroke dystonia. By highlighting some important knowledge gaps, we further aim to encourage future collaborative research projects.

## Definition

According to a recent consensus update, “dystonia is a movement disorder characterized by sustained or intermittent muscle contractions causing abnormal, often repetitive, movements, postures, or both. Dystonic movements are typically patterned, twisting, and may be tremulous. Dystonia is often initiated or worsened by voluntary action and associated with overflow muscle activation [involuntary activation of muscles that are not required to perform a given movement]” [7]. Dystonia can be focal, segmental, multifocal, or generalized. Hemi-dystonia is defined as dystonia involving the ipsilateral arm and leg with or without affecting the face, neck, or trunk. The former classification into “primary” and “secondary” dystonia is currently discouraged. The new etiological classification now includes pattern of inheritance and nervous system pathology [7].

## Epidemiology

At present, no reliable epidemiological data of post stroke movement disorders in childhood are available. In adults, post stroke movement disorders in general are considered rare, affecting only 1 % of stroke patients [8].

**Fig. 1:**
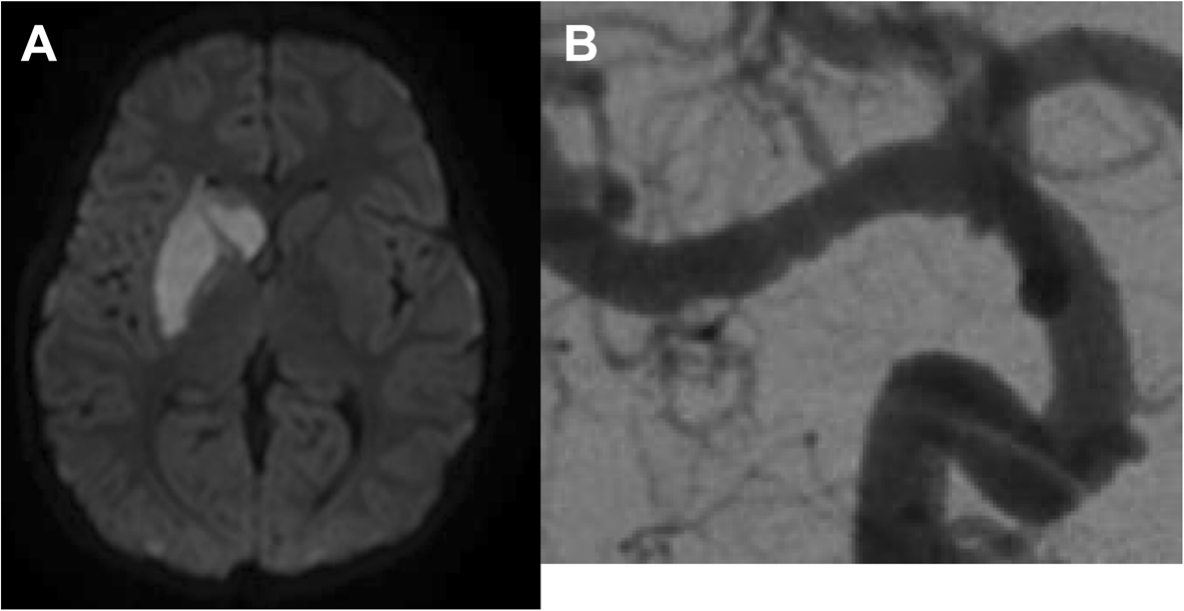
Right basal ganglia stroke (**a**) due to a focal cerebral arteriopathy involving the M1 segment of the right middle cerebral artery (MCA). Note the beading appearance of the affected MCA segment (**b**)

Post stroke-dystonia seems to be much more common among children than adults [6]. A Canadian study found that 21 % of the children with basal ganglia strokes will eventually develop dystonia [9]. This stroke pattern is particularly common in children with focal cerebral arteriopathy (FCA), an important, presumably inflammatory cause of CAIS (Fig. 1). FCA is a unilateral arteriopathy of the large vessels of the anterior circulation, typically affecting the distal ICA and proximal segments of the middle cerebral artery (MCA) and anterior cerebral artery (ACA) [10]. Cerebral infarcts due to FCA are nearly always located in perforator territories within the basal ganglia zone.

## Clinical characteristics and evaluation

Post stroke dystonia is usually unilateral, contralateral to the brain lesion; however, it can also be bilateral or generalized. Rarely, ipsilateral dystonia has been described [11]. Post stroke dystonia mostly involves the distal upper limb and often severely impairs manual functions [6, 12]. It can occur immediately at stroke onset (rare) or, typical in children, can be delayed by months or even years [13]. This is in contrast to post stroke spasticity, which typically evolves within the first few days or weeks post stroke. The reason for the delay of clinical manifestation is incompletely understood. Age-related maladaptive neuroplasticity as well as different CNS structures and networks involved is likely to play a role. Future functional imaging studies will help to better understand the underlying network damages.

Assessing dystonia is challenging because of its dynamic nature and overlap with other types of movement disorders [14]. One study on motor impairment after childhood onset stroke found that 15 of 24 (62.5 %) children with hemiparesis after stroke had additional dystonia [15]. Diagnosing dystonia in these cases is challenging; however, diagnostic instruments to assist with the differentiation between spasticity and dystonia are available [16]. These instruments enable to distinguish between involuntary movements or greater tone with purposeful movements (dystonia) or increased resistance during fast stretch compared to low stretch (spasticity).

Assessments capturing the impact of dystonia on motor function, activities of daily living, and the child’s ability to participate have also been invented [7]. Additional kinematic analyses (joint position, velocity, and acceleration of motion) can add important quantitative information about the functional severity more objectively [7, 17].

## Treatment dilemmas

Hemi-dystonia almost always has severe impact on motor function; however, response to treatment is typically poor [17]. Pharmacotherapy for dystonia is limited by lack of effect, especially in the long-term treatment, or because side effects are not tolerated ([17] Table 1). Botulinum toxin A injections in affected muscles can reduce painful spasms but do usually not directly improve function [17, 18]. Meanwhile, physiotherapy and occupational therapy are accepted as essential components to a multidisciplinary therapy; research in this field is particularly limited. Constraint induced movement therapy (CIMT), an established rehabilitation strategy after adult stroke and for unilateral cerebral palsy [19, 20], has not yet been sufficiently evaluated in children with dystonia. A single pilot study evaluated the effect of CIMT in six children after stroke, including three with dystonia, with positive effects on functional performance [21].

**Table 1:** Current and emerging treatment options for dystonia [16, 17]

Oral	- Anticholinergics (e.g., trihexyphenidyl) - Tetrabenazine - l-dopa/carbidopa - Baclofen - Benzodiazepines (e.g., diazepam, clonazepam) - Muscle relaxants (e.g., clonidin) - others (e.g., amitriptyline, gabapentin)
Intramuscular	- Botulinum toxin
Intrathecal	- Baclofen
Behavioral	- Biofeedback - Constraint induced therapy
(Noninvasive) neuromodulation	- Transcranial magnetic stimulation
(Invasive) neuromodulation	- Deep brain stimulation

Therefore, more studies and further treatment options are urgently needed.

Recently, biofeedback and transcranial magnetic stimulation (TMS) have gained some attention as noninvasive dystonia therapies. TMS is a painless method to stimulate the human brain and modulate its excitability [22]. Encouraging data from patients with writer’s cramp and the noninvasiveness of this technique make TMS an attractive candidate for future clinical studies in childhood [22].

Biofeedback studies focusing on inappropriate muscle activation and force adoption suggest that visual biofeedback of muscle activity can help to reduce excess muscle activation [23]. Children with dystonia may therefore to some extent be able to control co-contraction and reduce overflow by these strategies [17].

Deep brain stimulation (DBS) of the globus pallidus internus can be an effective and safe treatment option for inherited isolated generalized, segmental, and cervical dystonia with improvement in the Burke-Fahn-Marsden Dystonia Rating Scale (BFMDRS) of up 60–90 % [24], whereas the outcome for patients with acquired dystonia such as dyskinetic cerebral palsy is less distinctive and more heterogeneous, with a mean improvement of about 24 % [25]. The experience with uni- or bilateral DBS for pediatric patients with structural lesions due to stroke or traumatic brain injury is very limited to a few small case series [26, 27]. It is of note, that despite little or missing improvement in motor scores, patients report about other beneficial effects such as reduction of pain or decrease of muscle tone [27]. Therefore, despite little evidence to support this therapeutic option, DBS should be considered for young patients with disabling dystonia in the absence of other effective treatment options.

Future studies on greater (pediatric) cohorts are urgently needed to evaluate the effects of DBS in acquired forms of dystonia such as post stroke dystonia comprehensively and to identify prognostic markers for the therapeutic effect.

## Functional anatomy of basal ganglia

The pathophysiology of dystonia is complex and incompletely understood.

Commonly assumed models of basal ganglia dysfunction in dystonia are certainly oversimplified. However, they help to understand some basic ideas behind pathophysiology and also treatment.

In the majority of cases of post stroke dystonia, lesions in the basal ganglia, especially putamen, are found [11]. However, multiple other stroke locations have been associated, including thalamus, caudate, internal capsule, brainstem, cerebellum, and spinal cord [11]. Recent findings from functional imaging studies suggest that dystonia does not result from a single lesion but rather network dysfunctions and abnormalities in functional connectivity [28, 29]. Meanwhile, the basal ganglia and related thalamo-cortical networks are major determinants in the pathophysiology, sensorimotor cortex, and alterations of cerebello-thalamo-cortical pathways likely play an additional important role [28, 29].

Different resulting pathophysiological alterations have been suggested:

1. Defects of inhibitory circuits at the spinal, brainstem, cerebellar, or cortical level with decreased inhibition of unwanted muscle patterns
2. Abnormal sensory function and sensorimotor integration
3. Maladaptive plasticity of the sensorimotor cortex

However, it is increasingly recognized that different forms of dystonia have a different neuroanatomical origin, and results of recent electrophysiological and functional imaging studies of dystonia are not always consistent [28, 29]. Very few patients with post stroke dystonia have been studied. It is yet not clear to what extent pathophysiology of idiopathic or genetic and post stroke dystonia share common characteristics at network level [30–32].

After CAIS, it can be assumed that network changes differ significantly depending on the age at the time of the stroke event. Maladaptive plasticity may play a major role as animal studies of motor function outcome after stroke suggest that effects of plasticity can impair motor recovery by leading to network dysfunctions [33].

## Cerebral networks and modern imaging: missed opportunities?

With the progress of modern brain imaging techniques more detailed analyses of type, localisation and extent of ischemic lesions as well as cerebral reorganization and functional consequences after cerebral ischemia have been made possible. Along with the improved understanding of network dysfunctions, early prediction of dystonia and more specific treatment approaches may be available in the future. Nevertheless, comparing the enormous number of functional imaging studies in adults with (mostly inherited isolated) dystonia with the very limited research in children highlights obvious difficulties and challenges of studying children (Table 2). Progress in understanding the nature of childhood dystonia lacks far behind adult onset CNS diseases.

**Table 2:** Some pediatric challenges in functional imaging studies [28]

Small patient numbers	
Limited normative data	
Child unfriendly environment (MRI)	
Acoustic noise	
Motion artifacts	
Effects of maturation	
Effects of sedation	
Limited cooperation	
Monitoring task performance	
Limited acquisition time	

An increasing variety of (functional) imaging methods is available. Recently, resting state (rs) fMRI, the examination of spontaneous brain function by using blood oxygen level-dependent contrast in the absence of a task, has facilitated noninvasive mapping of neural network dysfunction even in children [34]. Using spontaneous activity, resting state maps can be generated reflecting functional brain organization. Limited rs fMRI data is available on dystonia [35, 36]. Abnormal functional connectivity was found in patients with writer’s cramp as well as cervical dystonia. Preliminary data suggest that this technique can be successfully used in disabled, asleep, or even sedated children with scanning times of about 5 min [34]. 

Magnetoencephalography (MEG) might be another noninvasive method for network studies in children. MEG is a technique that records magnetic fields generated by the brain. Because it is also possible to do depth recording, three-dimensional information can be gathered. MEG studies in patients with focal dystonias show considerable overlap with findings from neuroimaging studies indicating reduced inhibition and disturbed sensory-motor integration [37]. Future research will be needed to proof its reliable use in children.

## Conclusion and future directions

Dystonia after CAIS is a common yet insufficiently understood and poorly studied clinical challenge. Population-based studies are needed to better define prevalence, clinical presentation, time course, and treatment response of pediatric post stroke dystonia. Studies using modern, innovative imaging techniques will help to improve our understanding of the underlying pathophysiology and consequently the development of instruments for early prediction as well as targeted treatment of dystonia. Finally, treatment studies should evaluate whether noninvasive treatments such as TMS or biofeedback have the potential to improve motor function and quality of life in children with post stroke dystonia.
